# Exploring Cat–Human Interaction as a Psychosocial Resource in Autism and ADHD: Risks, Engagement, and Well-Being

**DOI:** 10.3390/bs16020162

**Published:** 2026-01-23

**Authors:** Lily Widdison, Ana Maria Barcelos, Stamatina Tsiora, Andrei Zarie, Daniel S. Mills, Niko Kargas

**Affiliations:** 1 School of Psychology, Sport Science and Wellbeing, University of Lincoln, Lincoln LN6 7TS, UK; lily.widdison@outlook.com; 2 School of Natural Sciences, University of Lincoln, Lincoln LN6 7TS, UK; abarcelos@lincoln.ac.uk (A.M.B.); dmills@lincoln.ac.uk (D.S.M.); 3 School of Social Sciences, Nottingham-Trent University, Nottingham NG1 4FQ, UK; andrei.zarie@ntu.ac.uk; 4 Department of Cognitive Sciences, United Arab Emirates University, Al Ain P.O. Box 15551, United Arab Emirates

**Keywords:** cat–human interaction, neurodiversity, autism, ADHD, mental health, pet–human relationships

## Abstract

Animals may offer vital psychosocial support, particularly for neurodiverse individuals. However, evidence surrounding the effects of pet ownership remains equivocal, especially in relation to cat–human dynamics. This study explored the relationship between cat–human-related factors (CHRFs) and psychological well-being in a sample of 127 adults, including individuals formally diagnosed with autism (30), ADHD (15), and/or co-occurring autism and ADHD (AuADHD; 22). Participants completed measures assessing neurodiverse traits, CHRF engagement, and symptoms of anxiety, depression, and suicidality. Spearman’s correlations analysed the relationships between CHRFs, neurodiverse traits, and well-being. Kruskal–Wallis tests established group differences in well-being and engagement in CHRFs between individuals with and without neurodevelopmental differences. The findings confirmed that autistic and ADHD traits were positively associated with greater anxiety, depression, and suicidality. Autistic individuals reported significantly elevated anxiety and depression; co-occurring diagnoses (AuADHD) were associated with heightened anxiety. Neurodiverse and neurotypical individuals demonstrated similar patterns of CHRF engagement. Several CHRFs, such as anxious cat behaviour, inability to provide for the cat, poor cat health, and close proximity, were linked to negative well-being outcomes. These findings highlight the nuanced, bi-directional nature of cat–human interactions, underscoring the importance of mitigating negative relational factors to support mental health in neurodiverse populations.

## 1. Introduction

### 1.1. Psychosocial Benefits of Companion Animals

Human–animal relationships may have the potential to improve social, emotional, and physical well-being (Amiot & Bastian, 2015; Beetz et al., 2012; Gee et al., 2021). For instance, psychosocial benefits of companion animals, often referred to as the ‘pet effect’ (Allen, 2003; Smith, 2012), are well-documented in the literature (Barker & Wolen, 2008; Friedmann & Son, 2009). The presence of a companion animal has been associated with an increase in positive affect (Janssens et al., 2020) such as an enhancement in the owners’ self-esteem (McConnell et al., 2011), self-acceptance (Merkouri et al., 2022), self-confidence (Zilcha-Mano et al., 2012), quality of life (Hughes et al., 2020), and life-satisfaction (Bao & Schreer, 2016). Additionally, a companion animal’s presence has been linked to a decrease in negative affect (Janssens et al., 2020), protecting against high levels of stress (Sugawara et al., 2012; Teo et al., 2022), anxiety (Ein et al., 2018), depression (Pereira & Fonte, 2018; Souter & Miller, 2007), and potentially suicidality (Hawkins et al., 2021; Love, 2021). These effects may have the ability to improve an individual’s daily functioning by enhancing social-emotional skills (Li et al., 2023; Purewal et al., 2017), which could aid the management of various mental (Brooks et al., 2018; Wisdom et al., 2009) and physical health conditions (Carr et al., 2018; Janevic et al., 2019).

In this context, the present study focuses on adult cat owners with and without formal diagnoses of autism and/or ADHD. Building on pet–human-related factor frameworks that distinguish specific supportive and strain-related activities and circumstances (Barcelos et al., 2020, 2023a; Corrêa et al., 2021; Ravenscroft et al., 2021), we examine how everyday cat–human-related factors (CHRFs) over a seven-day period relate to symptoms of anxiety, depression and suicidality. We further investigate whether these associations differ between neurodivergent and non-neurodivergent adults, in light of robust evidence that autistic and ADHD adults experience elevated and often chronic levels of anxiety, depression, and suicidality (Capp et al., 2024; Hedley & Uljarević, 2018; Hollocks et al., 2019; Lai et al., 2019; Sun et al., 2024; Zahid & Upthegrove, 2017). By integrating the pet–human-related factors framework with the neurodiversity and mental health literature, this study aims to clarify when and for whom cat–human relationships may act as psychosocial resources versus sources of strain. To explain the potential beneficial effects of human–animal interactions, a variety of psychosocial theories have been proposed (Barcelos et al., 2023b; Serpell et al., 2017). For example, the biophilia hypothesis posits that human beings have an intrinsic attraction toward living and life-like beings (Wilson 1984, as cited in Gullone, 2000). This inherent predisposition is evidenced towards animals (DeLoache et al., 2011), and it is argued that the simultaneous increase in attention results in decreased physiological arousal, which may result in a sense of security and safety (Beetz, 2017; Julius et al., 2013). This hypothesis may explain the benefits of close proximity with companion animals (Barcelos et al., 2023b). By contrast, the social support hypothesis asserts that companion animals provide and facilitate various forms of social support (Barcelos et al., 2023b; O’Haire, 2010). This includes emotional support via emotional contagion (Van Bourg et al., 2020; Yong & Ruffman, 2014), instrumental support via physical and psychological assistance (Burrows & Adams, 2008; Schoenfeld-Tacher et al., 2017), and positive social interaction via companionship (Horowitz & Hecht, 2016) and engagement from other human beings (Wood et al., 2015). These hypotheses should be understood as complementary and are not exclusive, with little evidence available to tease out the relative importance of differing mechanisms in general or in different populations (Barcelos et al., 2023b).

### 1.2. Inconsistent Findings in the Pet–Human Literature

Although a greater amount of published research concludes a positive relationship between pet ownership and human well-being (Barker & Wolen, 2008; Friedmann & Son, 2009), the conclusions remain contested due to potential publication bias of positive results (Herzog, 2011) and less attention given to studies showing no evidence of an effect or negative effects on psychological well-being (Scoresby et al., 2021; Wells, 2019). Contradictory findings exist regarding the effects of companion animals on stress, anxiety, and depression (Barker et al., 2018; Bradley & Bennett, 2015). Due to the mixed findings, it is argued that the general claim that a pet will improve mental health is unjustified (Herzog, 2011). There is undoubtedly a need for further research to strengthen any claims of psychosocial benefits, especially in specialist populations (Carr et al., 2020; Lisk et al., 2021). Variability in well-being outcomes in different populations is likely due to the complex and dynamic nature of human–animal interactions (Rodriguez et al., 2021). For example, it is argued that the notion of “pet ownership” effects is too nebulous, failing to recognise the heterogeneity of pet–human dyads (Barcelos et al., 2020), and features of pet–human relationships that may differentially influence well-being (Bao & Schreer, 2016; Janssens et al., 2020). There are also probably bi-directional effects, dependent on the context of the interaction (Barcelos et al., 2023a). Therefore, it is recommended that researchers adopt an approach that acknowledges the multifaceted nature of pet–human relationships (Gee & Mueller, 2019), instead of merely comparing pet owners with non-pet owners (Friedmann & Krause-Parello, 2018).

### 1.3. Pet–Human-Related Factors Framework

A framework of pet–human-related factors that define the nature of ownership has since been developed, encompassing a wide range of common activities and circumstances (Barcelos et al., 2023a) that may be direct or indirect, as well as active, passive, or neutral. This framework is based on initial qualitative research, across various samples, documenting how pet–human factors were reported to impact the well-being of owners (Barcelos et al., 2020, 2021a, 2021b; Corrêa et al., 2021; Ravenscroft et al., 2021). Greater eudaimonic and hedonic well-being have been reported by individuals who engage in general routine, shared outdoor activity and exercise, and tactile interactions with their dog (Barcelos et al., 2020, 2021a; Corrêa et al., 2021). However, only friendly conversations with others due to the dog and psychological closeness to the dog appear to be significantly associated with greater eudaimonic and hedonic well-being (Barcelos et al., 2021a, 2023a). Decreased eudaimonic and hedonic well-being have been consistently reported by individuals who experienced an inability to meet their dog’s needs, witnessed problematic dog behaviours, or had a dog of old age and poor health (Barcelos et al., 2020, 2023a, 2021a; Corrêa et al., 2021). Notably, failure to provide for the dog and problematic dog behaviour, such as displaying aggression and fear, have been associated with greater anxiety and depression (Barcelos et al., 2023a).

Although our understanding of the effects of dog–human-related factors is evolving, research investigating the effects of cat–human-related factors (CHRFs) on well-being is much more limited (Ravenscroft et al., 2021). The majority of research has failed to consider the relevance of the sort of factors identified in dogs on cat-owner well-being (e.g., Turner, 2017). For instance, cat owners are unlikely to relate to the psychosocial benefits associated with increased walking (Howell et al., 2017). The original qualitative framework by Barcelos et al. (2020) assessing the impact of dog–human-related activities was adapted for cat–human relationships by Ravenscroft et al. (2021). This qualitative study indicated that there may also be positive effects exclusive to cat–human relationships, such as a cat’s purring, which was reported to provide feelings of relaxation (Ravenscroft et al., 2021). An increase in eudaimonic and hedonic well-being has also been reported regarding feeding the cat, tactile interactions with the cat, and close proximity to the cat (Ravenscroft et al., 2021). On the other hand, problematic cat behaviour and poor cat health have been reported to decrease eudaimonic and hedonic well-being. Further empirical research is required to test the significance of these potential associations.

### 1.4. Neurodiversity and Well-Being

Autism spectrum disorder is characterised by atypical patterns of social communication and interaction across various contexts, as well as restricted and repetitive behaviours, interests, and activities (APA, 2022). Identity-first language is used to refer to autistic individuals, as it is broadly favoured by this population (Kenny et al., 2016; Taboas et al., 2023). Despite the substantial growth in the prevalence of recognised autistic individuals over the past 30 years (Solmi et al., 2022), social awareness and acceptance of the condition remain lacking (Sasson et al., 2017). The support needs of individuals on the spectrum are often inadequately met, as a result (Griffith et al., 2011; Raymaker et al., 2017). Autistic traits have been associated with greater rates of anxiety (Jolliffe et al., 2023), depression (Rai et al., 2018), and suicidality (Cassidy et al., 2018). Furthermore, individuals with a diagnosis of autism experience significant psychosocial challenges in adulthood (Howlin & Magiati, 2017), such as elevated rates of co-occurring mental health conditions and psychiatric disorders, notably anxiety and depressive disorders (Hollocks et al., 2019; Lai et al., 2019) and suicidality (Hedley & Uljarević, 2018; Hirvikoski et al., 2016; Zahid & Upthegrove, 2017). Attention-deficit hyperactivity disorder (ADHD) is prevalent in a significant proportion of autistic individuals (Rong et al., 2021).

ADHD is marked by persistent patterns of inattention, hyperactivity, and impulsivity that are inappropriate to the developmental level and inhibit an individual’s ability to function across multiple contexts (APA, 2022). These traits must have been present prior to age 12, and the majority of cases diagnosed in childhood persist into adulthood (Sibley et al., 2022). Adult ADHD has a diverse range of clinical presentations, with symptoms possibly going unrecognised (Song et al., 2021; Zalsman & Shilton, 2016). Additionally, due to a lack of recognition of ADHD as a lifelong disability, numerous individuals with ADHD do not receive the correct diagnosis or intervention (Kooij et al., 2018). Traits of ADHD have been associated with greater levels of anxiety and depression (Capp et al., 2024; Garcha & Smith, 2023) and suicidality (Sun et al., 2024). Furthermore, individuals with a diagnosis of ADHD experience a range of difficulties in psychosocial functioning (Agnew-Blais et al., 2018; Fayyad et al., 2016), for instance, increased rates of mental health conditions and co-occurring psychiatric disorders, especially anxiety and depressive disorders (Choi et al., 2022), as well as self-injurious behaviour and suicidality (Hinshaw et al., 2012; Septier et al., 2019).

Given the potential psychosocial benefits of pet ownership, pet–human relationships could provide an important source of support for neurodiverse individuals (Barcelos et al., 2021b). In autistic children, companion animals may improve social–emotional functioning (Byström & Persson, 2015; Carlisle et al., 2020; Harwood et al., 2019) and reduce levels of stress and anxiety (Carlisle, 2014; Wright et al., 2015). Although research into the effects of companion animals has overwhelmingly focused on autistic children, pet ownership has also been associated with enhanced mental well-being and social–emotional functioning in autistic adults (Atherton et al., 2023a; Barcelos et al., 2021b). The pet–human-related factors framework has previously been applied to autistic dog-owners (Barcelos et al., 2021b). In this case, individuals reported that close proximity with the dog and routine-like activities were beneficial for well-being and life functioning, protecting against suicidal tendencies in a sizeable minority (Barcelos et al., 2021b), whereas problematic dog behaviour, poor dog health, and obligations to the dog were reported to be detrimental to well-being. The effect of pet–human relationships on the well-being of ADHD individuals is much more limited by comparison (Schuck & Childress, 2023), although there is some evidence supporting the use of structured animal-assisted interventions in this population (Busch et al., 2016). Structured animal-assisted activities are typically goal-directed and designed to provide psychological support and enhance human well-being. Such interventions often involve trained and certified animals under the supervision of a professional handler (Kerulo et al., 2020). Further research is required to understand neurodiverse individuals’ engagement in pet–human-related factors and effects on their well-being, especially regarding ADHD individuals. This understanding could be used to strengthen the use of companion animals as a psychosocial intervention to maximise the well-being of individuals (Barcelos et al., 2023a).

Together, these lines of evidence indicate that autistic and ADHD adults face a substantial and often chronic burden of co-occurring anxiety, depression, and suicidality (Capp et al., 2024; Hedley & Uljarević, 2018; Hollocks et al., 2019; Lai et al., 2019; Sun et al., 2024; Zahid & Upthegrove, 2017), while frequently encountering barriers to timely diagnosis and appropriate mental health care (Griffith et al., 2011; Kooij et al., 2018; Pilling et al., 2012; Raymaker et al., 2017). Pet–human relationships have been proposed as a potentially accessible form of psychosocial support for neurodiverse individuals (Atherton et al., 2023a; Barcelos et al., 2021b; Schuck & Childress, 2023), yet existing empirical work has concentrated largely on autistic children and dog–owner relationships (Byström & Persson, 2015; Carlisle et al., 2020; Wright et al., 2015). The pet–human-related factors framework provides a structured approach to capturing both supportive and burdensome dog-related activities and circumstances (Barcelos et al., 2020, 2023a; Corrêa et al., 2021), and qualitative work suggests that analogous cat–human-related factors may similarly influence eudaimonic and hedonic well-being, for example, through feeding, tactile interaction, and close proximity to the cat (Ravenscroft et al., 2021). However, quantitative applications of this framework to everyday cat–human interactions in autistic and ADHD adults are lacking. Addressing this gap, and clarifying whether specific cat–human-related factors (CHRFs) function as psychosocial resources or sources of strain for neurodivergent adults compared with non-neurodivergent cat owners, is therefore an important step towards understanding when and for whom companion animals may support mental health.

### 1.5. The Present Study: Research Questions and Hypotheses

Building on this literature, the present study focused on adult cat owners with and without formal diagnoses of autism and/or ADHD. We applied a cat-adapted pet–human-related factors framework (Barcelos et al., 2023a; Ravenscroft et al., 2021) to examine how everyday cat–human-related factors (CHRFs) are associated with anxiety, depression, and suicidality over a seven-day period in these groups. Our overarching research question was the following: How are autistic and ADHD traits, formal neurodevelopmental diagnoses, and specific cat–human-related factors associated with anxiety, depression, and suicidality in adult cat owners, and do these patterns differ between neurodivergent and non-neurodivergent adults? Prior work shows that both autistic and ADHD traits are associated with elevated anxiety, depression, and suicidality in community and clinical samples (Capp et al., 2024; Cassidy et al., 2018; Garcha & Smith, 2023; Jolliffe et al., 2023; Rai et al., 2018; Sun et al., 2024). On this basis, we formulated four hypotheses.

**H1** **(traits and well-being).** *Higher autistic traits and higher ADHD traits will each be positively associated with anxiety, depression, and suicidality.*

**H2** **(diagnostic group differences in well-being).** *Adults with a formal diagnosis of autism, ADHD, or co-occurring autism and ADHD (AuADHD) will report higher anxiety, depression, and suicidality than adults without a formal diagnosis, reflecting the elevated rates of mood and anxiety disorders and suicidal behaviour observed in these diagnostic groups (Choi et al., 2022; Hedley & Uljarević, 2018; Hinshaw et al., 2012; Hollocks et al., 2019; Lai et al., 2019; Septier et al., 2019; Zahid & Upthegrove, 2017).*

**H3** **(CHRFs and well-being).** *In line with prior work applying the pet–human-related factors framework to dog–owner and cat–owner relationships, we predict that a perceived inability to provide for the cat and problematic cat behaviours (aggressive, anxious, and destructive) will be positively associated with anxiety, depression, and suicidality, and that poorer cat physical health will also be linked to poorer owner well-being (Barcelos et al., 2020, 2023a; Corrêa et al., 2021; Ravenscroft et al., 2021). We further anticipate that the strength of these associations may vary across diagnostic groups.*

**H4** **(diagnostic group differences in CHRF engagement).** *Given the heightened mental health burden reported in autistic and ADHD adults (Capp et al., 2024; Choi et al., 2022; Hollocks et al., 2019; Lai et al., 2019), and evidence that some neurodivergent individuals use relationships with companion animals as a form of psychosocial support (Atherton et al., 2023a; Barcelos et al., 2021b), we predict that adults with autism, ADHD, or AuADHD will report greater engagement than adults without a formal diagnosis in CHRFs involving close shared interaction with the cat (e.g., affectionate physical contact and close physical proximity).*

Because this study formed part of a wider umbrella project on pet–human relationships, some analyses of associations between CHRFs and autistic/ADHD traits were treated as exploratory and are interpreted with appropriate caution given the number of tests conducted (Greenland et al., 2016).

## 2. Materials and Methods

### 2.1. Participants

Participants were recruited using purposive sampling followed by snowball sampling techniques, utilising online platforms such as social media websites (e.g., Instagram, Facebook, LinkedIn). Inclusion criteria required participants to be 18 years or older, to currently own at least one cat, and to self-report either the presence or absence of a formal diagnosis of autism and/or ADHD. Eligibility further required that individuals could read the study materials, provide informed consent, and complete the online questionnaire independently. In total, 127 participants completed the study (*N* = 127). Most responses (70.1%; *n* = 89) were from within the UK. Participants were aged between 18 and 68 years (Mdn = 28, IQR = 12). The gender distribution was 76.4% female (*n* = 97), 14.2% male (*n* = 18), 6.3% non-binary (*n* = 8), 2.4% transgender (*n* = 3), and 0.8% unspecified (*n* = 1). Just under half of the sample (47.2%; *n* = 60) reported no formal diagnosis of a developmental condition. The remaining participants reported a formal diagnosis of autism (23.6%; *n* = 30), ADHD (11.8%; *n* = 15), or co-occurring autism and ADHD (AuADHD; 17.3%; *n* = 22). Including adults without a formal diagnosis provided a comparison group to determine whether patterns of well-being and engagement in cat–human-related factors are specific to neurodivergent adults or reflect broader trends among adult cat owners, in line with previous work emphasising the need to understand both shared and distinct aspects of pet–human relationships in autistic and non-autistic populations (Atherton et al., 2023a; Barcelos et al., 2021b).

### 2.2. Materials

The Dog–Human-Related Factors Questionnaire was adapted from Barcelos et al. (2023a) to reflect feline-specific behaviours and interaction patterns, with adaptations informed by Ravenscroft et al. (2021) to create a cat-specific version. For example, each original item was evaluated for applicability to cat–human interactions. Items referencing largely dog-specific activities (e.g., walking and running routines) were removed or rephrased to reflect typical feline behaviours. Language was adjusted to ensure species-appropriate terminology (e.g., barking vs. vocalisation episodes). Adaptations were reviewed by three researchers experienced in feline behaviour. This resulted in 11 items that were most relevant to cat owners. The large version developed by Ravenscroft et al. (2021) was not adopted, as the reduced item set was deemed more appropriate to prevent participant fatigue/disengagement. The abbreviated version of this questionnaire was derived from the most frequently mentioned themes in Ravenscroft et al. (2021) that captured the frequency and nature of interactions and events between participants and their cat(s). Table 1 presents the positive and negative factors evaluated, along with their respective measurement scales. A similar scaling to that used by Barcelos et al. (2023a) was used, with most items assessed using a self-report frequency rating scale ranging from 0 to 35, over the preceding seven days (e.g., in the last seven days, how many times have you had friendly conversations with others due to the presence of your cat?). Exceptions included ‘close proximity to the cat,’ measured on a sliding scale (0–100%), and ‘physical health of the cat,’ assessed using a 7-point Likert scale ranging from ‘Excellent’ to ‘Extremely Poor.’ Internal consistency assessment produced a Cronbach’s alpha of α = 0.63, which is considered “reliable”, but marginally so (Cohen et al., 2018, p. 744). This is consistent with the multidimensional and bi-directional nature of the cat–human interaction factors assessed; i.e., activities (items of the scale) are related to some extent but not highly so, since individuals vary in the emphasis they give to different aspects of their interactions with cats, and thus the nature of the relationship which emerges differs as a consequence (Ines et al., 2021).

The Autism Spectrum Quotient-10 (AQ-10; Allison et al., 2012) assesses the frequency of autistic traits in the individual across 5 domains: social skills difficulties, routine behaviour, attention switching, imagination, and attention to detail. It is a short-form version of that of Baron-Cohen et al. (2001), revised to include 10 items rated on a 4-point Likert scale (‘Definitely Disagree’ to ‘Definitely Agree’). For example, rating ‘Agree’ or ‘Definitely Agree’ in response to ‘When I’m reading a story I find it difficult to work out the characters’ intentions’ scored 1 point. There is reverse coding for 6 of the items for instance, rating ‘Definitely Disagree’ or ‘Disagree’ on ‘I find it easy to ‘read between the lines’ when someone is talking to me’ scored 1 point. Scores range from 0 to 10, with each item scored as either 0 or 1. Higher total scores indicate a greater presence of autistic traits. A score of 6 or above, in individuals of typical intelligence, is considered indicative of a positive screening outcome and may warrant clinical referral for further assessment. A Cronbach’s alpha of α = 0.76 indicated the measure was reliable (Cohen et al., 2018, p. 744).

The Adult ADHD Self-Report Scale Screener (ASRS; Kessler et al., 2005) was employed to assess the frequency of ADHD-related traits over the preceding six months. Grounded in the diagnostic criteria outlined in the fourth edition of the *Diagnostic and Statistical Manual of Mental Disorders* (DSM-IV; APA, 1994), the ASRS evaluates symptomatology across three core domains: inattention, hyperactivity, and impulsivity. The first six items of the ASRS serve as a validated screening tool, demonstrating a high predictive accuracy (94.3%) for identifying individuals at risk for ADHD (Adler et al., 2006). These items are therefore considered effective for preliminary assessment. Higher total scores reflect a greater presence of ADHD traits, with a score of 4 or above indicating a positive screening outcome. An assessment of internal consistency produced a Cronbach’s alpha of α = 0.69, suggesting marginal reliability, just below the conventional preferred threshold of 0.70 for a scale measuring a unitary construct (Cohen et al., 2018, p. 744). This value may thus reflect the variability of presentation of signs of ADHD, hence the use of the scale as a preliminary screen, rather than diagnostic tool.

The Generalised Anxiety Disorder—7 (GAD-7; Spitzer et al., 2006) assessed the frequency of Generalised Anxiety Disorder (GAD) symptomatology during the past 2 weeks, based on DSM-IV criterion. It includes 7 items rated on a 4-point Likert scale, with a score calculated using scores of between 0 (not at all) and 3 (nearly every day) for each question. The greater the total score, the greater the severity of anxiety symptoms (5 = mild, 10 = moderate, and 15 = severe). A score of ≥10 was considered a positive screening for clinical levels of anxiety (Spitzer et al., 2006). A Cronbach’s alpha of α = 0.87 indicated the items being used to measure the construct of interest were highly reliable (Cohen et al., 2018, p. 744).

The Patient Health Questionnaire—9 (PHQ-9; Kroenke et al., 2001) assessed the frequency of depressive symptomatology during the past 2 weeks, based on the 9 DSM-IV criteria for depressive disorders. The short-form version is based on Spitzer et al. (1999), including 9 items rated on a 4-point Likert scale (‘Not at all’—‘Nearly every day’), which ask how often the participant has experienced a variety of problems. Response options range from 0 (‘not at all’) to 3 (‘nearly every day’). Participants’ total scores can range from 0 to 27. The greater the total score, the greater the severity of depressive symptoms (5 = mild, 10 = moderate, 15 = moderately severe, and 20 = severe). Hence, a score of ≥10 was considered a positive screening for clinical levels of depression. A Cronbach’s alpha of α = 0.86 indicated the scale content was highly reliable (Cohen et al., 2018, p. 744).

The Suicidal Behaviours Questionnaire—Revised (SBQ-R; Osman et al., 2001) was used to assess the frequency and nature of suicidality. The instrument comprises four items, each targeting a distinct dimension of suicidal behaviour: lifetime suicidal ideation and attempts, the frequency of suicidal thoughts over the past year, the threat of suicidal behaviour, and the perceived likelihood of a future suicide attempt. Together, these items provide a brief yet multidimensional measure of suicidality. The greater the total score, the greater the risk of suicidality (6 or lower = low risk, 7 to 10 = moderate risk, 11 or higher = high risk.). In the general population, a score of ≥7 indicates a positive screening for risk of suicidality. A Cronbach’s alpha of α = 0.79 demonstrated the reliability of the content of this scale for this factor (Cohen et al., 2018, p. 744).

### 2.3. Procedure

This study received a favourable opinion by the University of Lincoln Ethics Committee (UoL 2024_16619). Data were gathered through the online platform QuestionPro, which participants accessed through their own computers. A link to the study was provided in the recruitment text stating the eligibility criteria (18+ years of age). The Participant Information Sheet stated the study aims, anonymous and secure storage of data, websites of support organisations, and email addresses of key researchers for any questions or concerns. Both resources provided content warnings relating to items concerning mental health issues and suicidality. Participants then had to provide fully informed consent to proceed. Demographic information included age, gender, country of residence, formal neurodevelopmental diagnoses, and a variety of cat ownership questions.

### 2.4. Data Analysis

This study was part of a wider umbrella study investigating the interplay of pet–human factor activities, mental health, personality, and neurodiversity. For the present report, we focused specifically on cat–human relationships, neurodiversity, and well-being. Given the non-parametric nature of the data, we used Spearman’s rank-order correlations and Kruskal–Wallis tests with Bonferroni-corrected Dunn’s post hoc tests, as appropriate. To test H1 (traits and well-being), we calculated Spearman’s rho correlations between autistic traits (AQ-10), ADHD traits (ASRS), anxiety (GAD-7), depression (PHQ-9), and suicidality (SBQ-R).

To test H2 (diagnostic group differences in well-being), we conducted three Kruskal–Wallis tests comparing GAD-7, PHQ-9, and SBQ-R scores across the four diagnostic groups: Group 1—no formal diagnosis of autism or ADHD; Group 2—a formal diagnosis of autism; Group 3—a formal diagnosis of ADHD; and Group 4—co-occurring formal diagnoses of autism and ADHD (AuADHD). Significant omnibus effects were followed up with Bonferroni-corrected Dunn’s post hoc comparisons.

To test H3 (CHRFs and well-being), we examined Spearman’s rho correlations between each of the 11 CHRFs and the mental health measures (GAD-7, PHQ-9, SBQ-R). In addition, we explored associations between CHRFs and autistic/ADHD traits (AQ-10, ASRS) to characterise how patterns of interaction with cats relate to neurodiversity at a dimensional level. Given the number of CHRFs and outcomes examined, these analyses were considered partly exploratory, and *p*-values are interpreted with appropriate caution in line with recommendations concerning multiple testing and effect-size interpretation (Greenland et al., 2016).

To test H4 (diagnostic group differences in CHRF engagement), we ran 11 Kruskal–Wallis tests comparing engagement in each CHRF across the four diagnostic groups defined above, followed by Bonferroni-corrected Dunn’s post hoc tests where applicable.

To evaluate the adequacy of the sample size for these analyses, we conducted power calculations in G*Power 3.1.9.7 (Faul et al., 2007). For the correlational analyses (H1 and exploratory correlations between CHRFs and autistic/ADHD traits), assuming a two-tailed α = 0.05 and medium effect sizes (ρ ≈ 0.25–0.30), the available sample (N = 127) afforded power greater than 0.80. For the Kruskal–Wallis tests comparing the four diagnostic groups (H2 and H4), the corresponding parametric one-way ANOVA model indicated adequate power to detect medium-high-sized omnibus effects but more limited power for small effects and for post hoc pairwise contrasts, particularly in the ADHD-only group (n = 15). These considerations align with our emphasis on effect sizes and cautious interpretation of *p*-values in the Results and with the limitations noted in Section 4.5 (Greenland et al., 2016).

## 3. Results

Results are organised by the four hypotheses outlined in Section 1.5. Section 3.1 addresses H1 (traits and well-being), Section 3.2 addresses H2 (diagnostic group differences in well-being), Section 3.3 addresses H3 (CHRFs and well-being), and Section 3.4 addresses H4 (diagnostic group differences in CHRF engagement).

### 3.1. Neurodiverse Traits and Well-Being (H1)

In relation to the first hypothesis (H1), correlations between autistic traits (AQ-10), ADHD traits (ASRS), anxiety (GAD-7), depression (PHQ-9), and suicidality (SBQ-R) are presented in Table 2, along with the descriptive statistics. There were moderate positive correlations between autistic traits and ADHD traits, *r_s_* = 0.44, *p* < 0.001, *n* = 125; autistic traits and anxiety, *r_s_* = 0.45, *p* < 0.001, *n* = 124; and autistic traits and depression, *r_s_* = 0.41, *p* < 0.001, *n* = 126. There was also a weak positive correlation between autistic traits and suicidality *r_s_* = 0.27, *p* < 0.001, *n* = 125. Additionally, there were moderate positive correlations between ADHD traits and anxiety, *r_s_* = 0.47, *p* < 0.001, *n* = 124, and ADHD traits and depression, *r_s_* = 0.49, *p* < 0.001, *n* = 125. There was also a weak positive correlation between ADHD traits and suicidality, *r_s_* = 0.33, *p* < 0.001, *n* = 124. Furthermore, there was a strong positive correlation between anxiety and depression, *r*_s_ = 0.75, *p* < 0.001, *n* = 124; a moderate positive corelation between anxiety and suicidality, *r_s_* = 0.54, *p* < 0.001, *n* = 124; and a moderate positive correlation between depression and suicidality, *r_s_* = 0.54, *p* < 0.001, *n* = 125. Overall, these findings support H1 and indicate that higher autistic and ADHD traits are associated with greater psychological distress in this sample.

### 3.2. Neurodevelopmental Diagnoses and Well-Being (H2)

**Figure 1:**
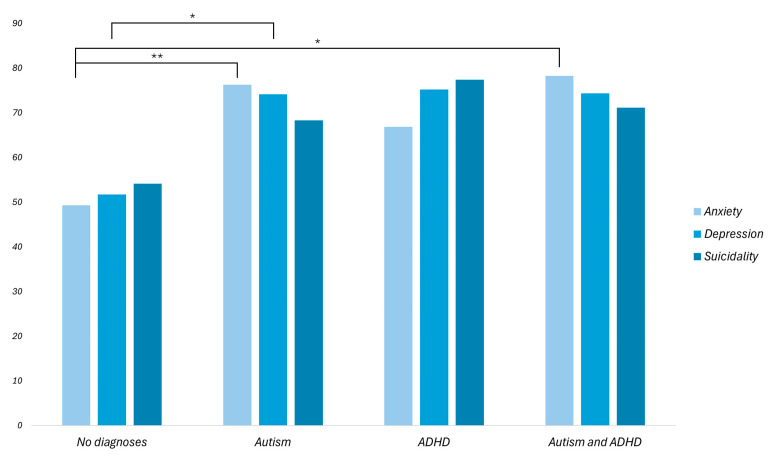
Mean ranks for anxiety, depression, and suicidality between individuals with and without formal diagnoses of autism and ADHD. Note: * *p* < 0.05. ** *p* < 0.01.

In relation to the second hypothesis (H2), group differences in GAD-7, PHQ-9, and SBQ-R scores across the four diagnostic groups (1—no formal diagnosis of autism or ADHD; 2—a formal diagnosis of autism; 3—a formal diagnosis of ADHD; 4—co-occurring formal diagnoses of autism and ADHD) are presented in Figure 1. In GAD-7, the differences between the mean ranks of 49.33 (Group 1, Mdn = 5.5), 76.30 (Group 2, Mdn = 9.5), 66.86 (Group 3, Mdn = 7), and 78.28 (Group 4, Mdn = 9) were significant, H (3, *n* = 124) = 16.62, *p* < 0.001. The mean rank of Group 2 was significantly higher than the mean rank of Group 1, *p* = 0.005. Furthermore, the mean rank for Group 4 was significantly higher than the mean rank for Group 1, *p* = 0.011. After adjustment for multiple testing, none of the other comparisons were significant.

In PHQ-9, the difference between the mean ranks of 51.73 (Group 1, Mdn = 6), 74.15 (Group 2, Mdn = 9), 75.20 (Group 3, Mdn = 10), and 73.57 (Group 4, Mdn = 9) were significant, H (3, *n* = 126) = 11.97, *p* = 0.007. The PHQ-9 mean rank for Group 2 was significantly higher than the PHQ-9 mean rank for Group 1, *p* = 0.036, with no other pairwise comparisons reaching significance after adjustment.

In SBQ-R, the difference between the mean ranks of 54.13 (Group 1, Mdn = 5), 68.30 (Group 2, Mdn = 7.5), 77.39 (Group 3, Mdn = 7.5), and 71.17 (Group 4, Mdn = 7) were not significant, H (3, *n* = 128) = 7.62, *p* = 0.054.

Taken together, these results provide partial support for H2 as autistic participants and those with co-occurring AuADHD reported significantly higher anxiety than participants without a formal diagnosis, and autistic participants reported higher depression, whereas suicidality did not differ significantly between diagnostic groups in the present sample.

### 3.3. Cat–Human-Related Factors and Well-Being (H3)

In relation to H3 (CHRFs and well-being), four CHRFs demonstrated weak but statistically significant relationships with the mental health measures (Table 3). Firstly, there were positive correlations between an inability to provide for the cat and depression, *r_s_* = 0.20, *p* = 0.028, *n* = 126, and an inability to provide for the cat and suicidality, *r_s_* = 0.19, *p* = 0.036, *n* = 125. Additionally, there were positive correlations between anxious cat behaviour and anxiety, *r_s_* = 0.26, *p* = 0.003, *n* = 124, and anxious cat behaviour and depression, *r_s_* = 0.25, *p* = 0.004, *n* = 126. There was a positive correlation between close proximity to the cat and anxiety, *r_s_* = 0.22, *p* = 0.013, *n* = 124. Finally, there was a positive correlation between poor physical health of the cat and depression, *r_s_* = 0.22, *p* = 0.015, *n* = 126. The other six CHRFs did not yield significant correlations with anxiety, depression, or suicidality. These results therefore provide partial support for H3, indicating that a subset of hypothesised strain-related CHRFs are associated with poorer mental health, whereas most CHRFs were not reliably related to distress in this sample.

### 3.4. Engagement in Cat–Human-Related Factors (H4)

In relation to H4 (diagnostic group differences in CHRF engagement), three CHRFs displayed significant differences in engagement between the four diagnostic groups. Firstly, for feeding the cat, the difference between the mean ranks of 61.11 (Group 1, Mdn = 9), 54.13 (Group 2, Mdn = 9), 84.83 (Group 3, Mdn = 16), and 71.14 (Group 4, Mdn = 12.5) were significant, H (3, *n* = 127) = 8.28, *p* = 0.041. The mean rank for Group 3 (ADHD) was significantly higher than the mean rank for Group 2 (autism), *p* = 0.047. None of the other comparisons were significant after adjustment for multiple testing. Secondly, for witnessing cat vocalisation episodes, the difference between the mean ranks of 58.13 (Group 1, Mdn = 12), 57.80 (Group 2, Mdn = 10.5), 76.03 (Group 3, Mdn = 32), and 80.25 (Group 4, Mdn = 24.5) were significant, H (3, *n* = 127) = 8.42, *p* = 0.038. However, post hoc pairwise comparisons using Dunn’s test with adjustment failed to identify specific significant pairwise contrasts. Finally, for affectionate physical contact with the cat, the difference between the mean ranks of 56.56 (Group 1, Mdn = 22), 63.95 (Group 2, Mdn = 32), 82.70 (Group 3, Mdn = 35), and 71.61 (Group 4, Mdn = 35) were significant, H (3, *n* = 127) = 7.86, *p* = 0.049, although again no individual pairwise comparisons remained significant following adjustment. The other eight CHRFs showed no significant differences in engagement between groups.

Overall, these findings indicate limited support for H4, as, although there were some group differences in feeding, vocalisation, and affectionate physical contact, engagement in most CHRFs did not differ significantly between neurodivergent and non-neurodivergent participants.

## 4. Discussion

The present study investigated the well-being of neurodivergent and non-neurodivergent adult cat owners in the context of everyday cat–human relationships over a seven-day period. In line with H1 and H2, higher autistic and ADHD traits were associated with greater anxiety, depression, and suicidality, and autistic and AuADHD groups reported elevated anxiety (and, for autistic adults, elevated depression) compared with adults without a formal diagnosis (Capp et al., 2024; Hedley & Uljarević, 2018; Hollocks et al., 2019; Lai et al., 2019; Zahid & Upthegrove, 2017). H3 received partial support as only a small subset of predominantly strain-related CHRFs (i.e., perceived inability to provide for the cat, anxious cat behaviour, close proximity to the cat, and poorer cat physical health) were associated with poorer mental health, whereas most CHRFs showed no reliable associations with distress (Barcelos et al., 2020, 2023a; Corrêa et al., 2021; Ravenscroft et al., 2021). H4 received only limited support as there were some group differences in feeding, cat vocalisation episodes, and affectionate physical contact, but engagement in most CHRFs did not differ significantly between neurodivergent and non-neurodivergent participants. Overall, these findings suggest that both autistic/ADHD traits and diagnoses are meaningfully associated with mental health in adult cat owners and that specific high-burden CHRFs are linked to poorer well-being, while patterns of everyday cat engagement are broadly similar across groups.

### 4.1. Neurodiverse Traits and Well-Being

The results support H1, which predicted significant positive correlations between autistic traits, ADHD traits, and poorer well-being (i.e., anxiety, depression, and suicidality). Individuals with higher levels of autistic and ADHD traits reported greater rates of anxiety and depression and were at increased risk of suicidality, mirroring previous trait-based findings in community and clinical samples (Capp et al., 2024; Cassidy et al., 2018; Garcha & Smith, 2023; Jolliffe et al., 2023; Rai et al., 2018; Sun et al., 2024). These data reinforce the view that autistic and ADHD traits, conceptualised dimensionally rather than categorically, have meaningful implications for mental health. This dimensional pattern is important because many individuals with substantial autistic or ADHD traits experience barriers to timely diagnosis and tailored intervention, including under-recognition in some demographic groups and limited access to specialist assessment (Griffith et al., 2011; Kooij et al., 2018; Pilling et al., 2012; Raymaker et al., 2017; South et al., 2020). Our findings therefore support calls for mental health support to be sensitive to trait load and functional impact rather than restricted solely to those with confirmed diagnoses (Landry & Chouinard, 2019). They are also consistent with prior work documenting substantial overlap in autistic and ADHD traits and shared or interacting symptom profiles (Ghirardi et al., 2019; Mayes et al., 2012; Panagiotidi et al., 2019; Taylor et al., 2015), and with evidence that co-occurring autism and ADHD is common (Rong et al., 2021). Extending this literature to adult cat owners indicates that the presence of a companion animal does not eliminate underlying vulnerabilities, in line with research suggesting that pets can support but do not fully buffer mental health difficulties in neurodivergent and other clinical populations (Atherton et al., 2023a; Barcelos et al., 2021b; Brooks et al., 2018; Hawkins et al., 2021).

### 4.2. Formal Diagnoses and Well-Being

H2 predicted that adults with formal diagnoses of autism, ADHD, or co-occurring autism and ADHD would report poorer well-being than adults without a diagnosis. This was partially supported. Anxiety and depression were significantly higher in autistic adults than in adults without a diagnosis, and anxiety was elevated in the AuADHD group, consistent with meta-analytic evidence of high rates of anxiety and depressive disorders and complex psychiatric comorbidity in autistic adults (Hollocks et al., 2019; Hossain et al., 2020; Lai et al., 2019). These findings add to concerns that mental health provision for autistic adults remains limited or poorly tailored (Pilling et al., 2012) and underscore the need to identify factors that may support or undermine well-being, including pet–human relations (Barcelos et al., 2021b; Brumpton & Kargas, 2026; Howlin & Taylor, 2015; National Collaborating Centre for Mental Health, 2012; Segers & Rawana, 2014). In contrast, adults with ADHD alone did not report significantly higher anxiety or depression than adults without a diagnosis, despite prior evidence of elevated mood and anxiety disorders in this group (Agnew-Blais et al., 2018; Choi et al., 2022; Fayyad et al., 2016). This likely reflects limited power for subgroup comparisons, given the relatively small ADHD-only group (n = 15), as also noted in our power analysis and Limitations (Faul et al., 2007; Greenland et al., 2016). Suicidality did not differ significantly between diagnostic groups, although the omnibus test approached significance, in line with broader work linking both autism and ADHD to elevated suicide risk (Hedley & Uljarević, 2018; Hinshaw et al., 2012; Septier et al., 2019; Zahid & Upthegrove, 2017). Overall, these findings indicate that poorer well-being in neurodivergent adults is not uniform across all diagnostic categories and all outcomes. Anxiety appears particularly elevated in autistic and AuADHD individuals, depression is especially elevated in autistic adults, and differences in suicidality may require larger samples to detect.

### 4.3. Cat–Human-Related Factors and Well-Being

Consistent with H3, a small number of cat–human-related factors were positively correlated with anxiety, depression, and suicidality. Perceived inability to provide for the cat was associated with greater depression and suicidality; anxious cat behaviour was associated with greater anxiety and depression; close proximity to the cat was associated with greater anxiety; and poorer cat physical health was associated with greater depression. These findings parallel dog-owner research using the pet–human-related factors framework, where caregiving strain, problematic animal behaviour, and concerns about the animal’s health have been linked to diminished well-being (Barcelos et al., 2020, 2021a, 2023a; Corrêa et al., 2021), and are consistent with evidence that chronic companion–animal health and behavioural problems can adversely affect owner affect and stress (Amat et al., 2016; Applebaum et al., 2020; Johnson et al., 2021; Ravenscroft et al., 2021). Although effect sizes were small, this pattern fits the expectation that everyday CHRFs make incremental contributions to mental health alongside many other psychosocial risk and protective factors (Greenland et al., 2016; Rodriguez et al., 2021). In contrast to some dog-owner studies, aggressive and destructive cat behaviours were not robustly associated with poorer well-being in the present sample (Barcelos et al., 2020, 2023a). Species-related differences may partly explain this discrepancy. For instance, companion dogs are more frequently referred for behavioural problems, and problematic feline behaviour often manifests as anxiety, withdrawal, and vocalisation rather than overt aggression (Denenberg et al., 2005; Howell et al., 2017). As such, caregiver strain in cat owners may arise more from managing ongoing anxiety-related issues and health concerns than from isolated incidents of aggression, in line with reports of owner stress associated with anxious or unwell cats (Amat et al., 2016; Applebaum et al., 2020; Johnson et al., 2021; Ravenscroft et al., 2021).

Notably, the positive association between close proximity to the cat and anxiety was unexpected given prior work indicating that psychological closeness and time spent in proximity to companion animals can support eudaimonic and hedonic well-being and buffer stress (Barcelos et al., 2021a; Ein et al., 2018; Ravenscroft et al., 2021; Sugawara et al., 2012; Teo et al., 2022). One plausible explanation is that more anxious owners may actively seek out physical closeness with their cat as a short-term regulatory strategy. Because our data are cross-sectional and rely on seven-day retrospective self-reports, we cannot determine directionality, that is, whether proximity increases anxiety or anxiety motivates proximity. Future longitudinal and experience-sampling designs will be needed to test these possibilities (Barcelos et al., 2023a).

Finally, the present study is, to our knowledge, the first to apply a pet–human-related factors framework quantitatively to cat–human relationships in a neurodiverse-inclusive adult sample (Barcelos et al., 2023a; Ravenscroft et al., 2021). In line with arguments from dog–human work, our findings suggest that intentionally reducing high-burden CHRFs, such as perceived inability to provide for the cat, or managing anxious or chronically unwell cats, may enhance the psychosocial benefits of cat ownership (Barcelos et al., 2021b). This has practical implications for collaborative work between mental health professionals and animal behaviour and welfare specialists, particularly given evidence that autistic adults may perceive barriers to successful pet care (Atherton et al., 2023a; Barcelos et al., 2024).

### 4.4. Engagement in Cat–Human-Related Factors

H4 predicted that adults with formal diagnoses of autism, ADHD, or co-occurring autism and ADHD would report greater engagement in CHRFs involving shared interaction (e.g., affectionate physical contact and close physical proximity) than adults without a diagnosis, in line with evidence that neurodivergent individuals often experience elevated distress and may seek psychosocial support through relationships with companion animals (Atherton et al., 2023a; Barcelos et al., 2021b; Choi et al., 2022; Hollocks et al., 2019). This prediction received only limited support as, for most CHRFs, no significant group differences in engagement were observed, suggesting that neurodivergent and non-neurodivergent adults engaged with their cats in broadly similar ways in everyday life.

Three CHRFs showed significant omnibus group differences (i.e., feeding the cat, cat vocalisation episodes, and affectionate physical contact). Individuals in the ADHD group fed their cats more frequently than autistic individuals, and neurodivergent participants tended to report somewhat higher levels of affectionate physical contact than non-neurodivergent participants, although post hoc contrasts were modest and often did not survive adjustment. Feeding has been linked to improved eudaimonic and hedonic well-being in cat owners (Ravenscroft et al., 2021), and it is possible that some individuals engage more often in caregiving routines when seeking positive affective states, though empirical evidence on human–animal interactions in ADHD remains limited (Busch et al., 2016; Schuck & Childress, 2023). Taken together with the largely null findings for other CHRFs, these results suggest that relationships with cats, as with dogs, may offer broadly comparable forms of interaction across neurodivergent and non-neurodivergent adults (Atherton et al., 2023b; Barcelos et al., 2023a, 2021a). In combination with the CHRF–well-being associations discussed above, this pattern implies that the quality and context of specific cat–human interactions may be more important for mental health than overall engagement frequency per se.

### 4.5. Limitations and Future Directions

Several limitations should be considered when interpreting these findings. First, formal diagnoses of autism, ADHD, and co-occurring autism and ADHD were self-reported and could not be independently verified. Although this approach is common in survey research, it introduces the possibility of misclassification and underscores the need for future work combining self-reports with clinical verification where feasible (Griffith et al., 2011; Pilling et al., 2012). Second, the ADHD-only group was relatively small (*n* = 15), which limited statistical power to detect between-group differences, particularly for depression and anxiety, and likely contributed to the near-significant omnibus effect for suicidality. Our power analysis indicated that the overall sample size was adequate for detecting medium-high-sized correlations and omnibus group effects, but not small effects or some subgroup contrasts (Faul et al., 2007; Greenland et al., 2016). Third, the sample was predominantly female and largely based in the UK, a pattern frequently observed in human–animal interaction research (Herzog, 2021) but one which limits the generalisability of the findings to more diverse populations. Fourth, all measures relied on self-reports of experiences over the past seven days. This permitted fine-grained, recent reflections on well-being and CHRFs but is inherently cross-sectional and retrospective, preventing strong inferences about temporal ordering and directionality (e.g., whether anxiety motivates increased proximity to the cat or vice versa). Future research should therefore apply this framework using longitudinal and experience-sampling designs and with larger, more representative samples to test the robustness and temporal dynamics of the associations reported here (Barcelos et al., 2023a; Rodriguez et al., 2021). Fifth, we examined multiple CHRFs in relation to several mental health and neurodiversity indices. Although we used Bonferroni-corrected Dunn tests for group comparisons, we did not formally correct for multiple testing in the correlation analyses. In line with contemporary recommendations, we therefore emphasise the pattern and plausibility of effects rather than any single *p*-value or threshold (Greenland et al., 2016), and future work could use multivariate approaches or dimension-reduction techniques to summarise CHRFs. Future studies could also broaden the range of outcomes examined. In particular, assessing positive well-being indicators (e.g., life satisfaction, meaning in life, and social connectedness) alongside distress could deepen understanding of the complex and bi-directional nature of cat–human relationships (Barcelos et al., 2023a; Rodriguez et al., 2021). It will also be important to explore whether neurodivergent individuals show species-specific preferences or derive differential benefits from different companion animals, given emerging evidence that dog ownership can support mental health and buffer suicidality in autistic adults (Barcelos et al., 2021b). Finally, potential mediating and moderating factors such as personality, attachment to the pet, and social support (Atherton et al., 2023a; Bao & Schreer, 2016; Miltiades & Shearer, 2011) warrant investigation to clarify when and for whom cat–human relationships buffer or exacerbate psychological distress.

## 5. Conclusions

Pet–human-related factors frameworks provide a structured way to consider the bi-directional impact of specific pet–human activities and circumstances on human well-being (Barcelos et al., 2023a). Applying this sort of framework may help resolve inconsistencies in previous work on pet ownership and mental health by capturing both the supportive and the burdensome aspects of caregiving (Scoresby et al., 2021; Wells, 2019). The present study extended this framework to cat–human relationships in neurodiverse individuals, addressing a notable gap in the literature on both neurodiversity and companion animals (Atherton et al., 2023a; Ravenscroft et al., 2021).

In line with H1 and H2, we found that autistic and ADHD traits were associated with reduced well-being and that autistic and AuADHD adults, in particular, reported elevated anxiety (and, for autistic adults, elevated depression) compared with adults without a formal diagnosis, reinforcing evidence of heightened distress in neurodiverse populations (Capp et al., 2024; Hedley & Uljarević, 2018; Hollocks et al., 2019; Lai et al., 2019; Zahid & Upthegrove, 2017). These findings emphasise the necessity of improving mental health support for autistic and AuADHD adults (Pilling et al., 2012) and of considering both dimensional traits and diagnostic status when assessing risk.

Consistent with H3, four CHRFs (i.e., perceived inability to provide for the cat, anxious cat behaviour, close proximity to the cat, and poorer cat physical health) were associated with negative well-being outcomes, paralleling prior work on dog–human-related factors (Barcelos et al., 2020, 2021a; Corrêa et al., 2021). These findings reiterate the bi-directional nature of pet–human-related factors, which has rarely been quantified in cat–human relationships (Ravenscroft et al., 2021), and suggest that reducing high-burden CHRFs may be a pragmatic target for interventions aiming to support neurodivergent cat owners.

Finally, H4 received only limited support. Although there were some group differences in feeding, vocalisation, and affectionate physical contact, overall engagement in CHRFs did not differ substantially between neurodivergent and non-neurodivergent individuals. This pattern suggests that relationships with cats, as with dogs (Barcelos et al., 2023a, 2021a), may offer broadly comparable forms of interaction across groups, rather than reflecting a uniformly heightened reliance on cats for support in neurodivergent adults.

Taken together, these findings underscore the importance of considering both neurodiversity and specific cat–human-related factors when evaluating the mental health of adult cat owners. They also highlight the potential value of collaborative work between mental health professionals and animal behaviour/welfare specialists in identifying and mitigating high-burden CHRFs, with the aim of maximising the psychosocial benefits of cat–human relationships while minimising associated risks. Future research with larger and more representative samples, and with longitudinal designs, will be essential to test the robustness and causal direction of the associations reported here.

## Figures and Tables

**Table 1:** Eleven cat–human-related factors assessed for their occurrence/rating during the past 7 days.

Factor	Response Options
Friendly conversations with others due to the presence of your cat	Zero (0)–35 or more (35) times overall
Feeding your cat main meals	Zero (0)–35 or more (35) times overall
Aggressive behaviour displayed by your cat (e.g., biting, lunging)	Zero (0)–35 or more (35) times overall
An inability to provide for your cat (e.g., not able to be at home with or feed them)	Zero (0)–35 or more (35) times overall
Anxious behaviour displayed by your cat (e.g., fear of people, separation anxiety)	Zero (0)–35 or more (35) times overall
Vocalisation episodes displayed by your cat	Zero (0)–35 or more (35) times overall
Destructive behaviour displayed by your cat (e.g., chewed, scratched, stolen items)	Zero (0)–35 or more (35) times overall
Affectionate physical contact with your cat (e.g., petting, cuddling, kissing)	Zero (0)–35 or more (35) times per day in the last 7 days
House soiling by your cat with urine or faeces (e.g., on inappropriate surfaces)	Zero (0)–35 or more (35) times overall
Proportion of time awake in close proximity with your cat (e.g., in the same room or outside together)	0–100% times overall
Physical health of your cat (e.g., medical issues, sickness, injuries)	Excellent (7), Very good (6), Good (5), Neither good nor poor (4), Poor (3), Very poor (2), Extremely poor (1)

Note: adapted from Barcelos et al. (2023a) with modifications to ensure relevance to cat–human interactions, including removal of dog-specific items, rewording for species-appropriate behaviours, and consideration of relevant items identified in Ravenscroft et al. (2021).

**Table 2:** Descriptive statistics and correlations for autistic traits, ADHD traits, anxiety, depression, and suicidality.

	*n*	Mdn	IQR	Autistic Traits	ADHD Traits	Anxiety	Depression	Suicidality
Autistic Traits	127	5	4	–				
ADHD Traits	125	4	3	0.44 **	–			
Anxiety	124	7	7	0.45 **	0.47 **	–		
Depression	126	8	8	0.41 **	0.49 **	0.75 **	–	
Suicidality	125	6	5.5	0.27 **	0.33 **	0.54 **	0.54 **	–

Note: ** *p* < 0.001.

**Table 3:** Correlations between cat–human-related factors, anxiety, depression, and suicidality.

	Anxiety	Depression	Suicidality
Social interaction due to the cat	0.06	0.10	0.03
Feeding the cat	0.01	−0.04	0.07
Aggressive cat behaviour	0.04	−0.01	−0.02
Inability to provide for the cat	0.11	0.20 *	0.19 *
Anxious cat behaviour	0.26 **	0.25 **	0.11
Cat vocalisation episodes	0.05	−0.05	0.07
Destructive cat behaviour	0.05	0.12	0.10
Affectionate physical contact with the cat	0.10	−0.02	0.17
Cat soiling in the house	0.06	0.13	0.09
Close proximity to the cat	0.22 *	0.17	0.17
Physical health of the cat	0.16	0.22 *	−0.01

Note. * *p* < 0.05. ** *p* < 0.01.

## Data Availability

The data presented in this study are available on request from the corresponding author.

## Abbreviations

The following abbreviations are used in this manuscript:

CHRFs	Cat–Human-Related Factors
APA	American Psychiatric Association
ADHD	Attention-Deficit Hyperactivity Disorder
